# Effect of Gut Microbiota and *PNPLA3* rs738409 Variant on Nonalcoholic Fatty Liver Disease (NAFLD) in Obese Youth

**DOI:** 10.1210/clinem/dgaa382

**Published:** 2020-06-19

**Authors:** Ayesha Monga Kravetz, Todd Testerman, Brittany Galuppo, Joerg Graf, Bridget Pierpont, Stephan Siebel, Richard Feinn, Nicola Santoro

**Affiliations:** 1 Frank H. Netter MD School of Medicine, North Haven, Connecticut; 2 Department of Pediatrics, Yale University School of Medicine, New Haven, Connecticut; 3 Department of Molecular and Cell Biology, University of Connecticut, Storrs, Connecticut; 4 Department of Medicine and Health Sciences, “V. Tiberio,” University of Molise, Campobasso, Italy

**Keywords:** NAFLD, gut microbiota, pediatrics, obesity

## Abstract

**Context:**

Nonalcoholic fatty liver disease (NAFLD) is the most common cause of liver disease, affecting approximately 3 in 10 obese children worldwide.

**Objective:**

We aimed to investigate the potential relationship between gut microbiota and NAFLD in obese youth, while considering the role of *PNPLA3* rs738409, a strong genetic contributor to NAFLD.

**Design:**

In this cross-sectional study, participants completed an abdominal magnetic resonance imaging to measure hepatic fat fraction (HFF), oral glucose tolerance test, and *PNPLA3* rs738409 genotyping. Fecal samples were collected to analyze the V4 region of the 16S rRNA gene for intestinal bacteria characterization.

**Setting:**

Yale Pediatric Obesity Clinic.

**Participants:**

Obese youth (body mass index >95th percentile) with NAFLD (HFF ≥5.5%; n = 44) and without NAFLD (HFF <5.5%; n = 29).

**Main Outcome Measure:**

Shannon-Wiener diversity index values and proportional bacterial abundance by NAFLD status and *PNPLA3* genotype.

**Results:**

Subjects with NAFLD had decreased bacterial alpha-diversity compared with those without NAFLD (*P* = 0.013). Subjects with NAFLD showed a higher Firmicutes to Bacteroidetes (F/B) ratio (*P* = 0.019) and lower abundance of Bacteroidetes (*P* = 0.010), *Prevotella* (*P* = 0.019), *Gemmiger* (*P* = 0.003), and *Oscillospira* (*P* = 0.036). F/B ratio, Bacteroidetes, *Gemmiger*, and *Oscillospira* were associated with HFF when controlling for group variations. We also observed an additive effect on HFF by *PNPLA3* rs738409 and *Gemmiger*, and *PNPLA3* rs738409 and *Oscillospira*.

**Conclusions:**

Obese youth with NAFLD have a different gut microbiota composition than those without NAFLD. These differences were still statistically significant when controlling for factors associated with NAFLD, including *PNPLA3* rs738409.

Nonalcoholic fatty liver disease (NAFLD) is the most common cause of liver disease in the pediatric population, affecting approximately 3 in 10 obese children worldwide (1). The disease is characterized by macrovesicular steatosis in more than 5% of hepatocytes in an individual without significant history of alcohol intake or other liver pathologies (2, 3). Notably, NAFLD drastically affects lipid and glucose metabolism, increasing the risk to insulin resistance and dyslipidemia early in life (4, 5). While the pathophysiology of NAFLD is unclear, genetic contributions and gut microbiota are considered to be important players.

One of the strongest genetic contributors of NAFLD is a variant in the Patatin-like phospholipase 3 gene (*PNPLA3*), a gene encoding for a protein called adiponutrin, which has been found to confer susceptibility to increased hepatic fat levels and hepatic inflammation (6). The *PNPLA3* rs738409 variant is a single-nucleotide polymorphism in which there is a change from cytosine to guanine that leads to the amino acid substitution of isoleucine to methionine at the 148 position of the coding sequence, in the active site of the enzyme (6, 7). Although the association between the *PNPLA3* gene and NAFLD has been known for almost a decade, the physiologic role of adiponutrin and the mechanisms by which the rs738409 variant predisposes to NAFLD remain unclear (8, 9). *PNPLA3* rs738409 has been observed to confer predisposition to hepatic steatosis in obese youth without increasing the level of insulin resistance (10). Allele frequency of the *PNPLA3* rs738409 risk allele is lower in African Americans, thus African American obese youth possess some protection from liver steatosis (11, 12). 

Gut microbiota dysbiosis has been associated with obesity and, most recently, NAFLD (13, 14). This association has been attributed, in part, to disruption of gut epithelial tight junctions through which bacteria and toxins gain access to the portal circulation and release inflammatory cytokines that promote hepatic steatosis and inflammation (15). To our knowledge, only 3 studies have investigated the gut microbiota composition in obese children with and without NAFLD, which controls for the obesity component that is associated with gut microbiota dysbiosis. Del Chierico et al (16) observed increased hierarchy of ecological diversity in obese pediatric patients compared with pediatric patients with nonalcoholic fatty liver (NAFL). The researchers did not find significant differences between the 2 groups in the bacterial abundance at the species and genus levels. In contrast, Michail et al (17) noted that obese children with NAFLD had more abundant Gammaproteobacteria and *Prevotella* compared with obese children without NAFLD. In the Michail et al study, no difference between the groups, in Firmicutes and Bacteroidetes or their ratio, was detected. Firmicutes and Bacteroidetes have become 2 phyla of interest after Gordon and colleagues discovered that Firmicutes abundance increases while Bacteroidetes abundance decreases in obese subjects, as compared with lean subjects (18, 19). Schwimmer et al (20) noted an association between NAFLD and intestinal dysbiosis. The researchers observed lower alpha diversity in children with NAFLD compared with the control group and more severe fibrosis in children with higher abundance of *Prevotella copri*. These previous studies that assessed the effect of microbiota on NAFLD did not account for the role of *PNPLA3*. In our study, we aimed to explore the potential relationship between gut microbiota and NAFLD in a group of obese youth, while also considering the role of *PNPLA3* rs738409.

## Methods

### Study cohort

A total of 73 children and adolescents with obesity (body mass index [BMI] ≥95th percentile) were recruited from the Yale Pediatric Obesity Clinic. All participants completed an oral glucose tolerance test (OGTT) to assess glucose metabolism and a fast-magnetic resonance imaging (MRI) abdominal scan to assess body fat partitioning and intrahepatic fat content. In addition, fasting blood samples were collected at the OGTT visit to obtain liver function tests, a lipid panel, and a genetic test. Metabolic studies were conducted at the Yale Center for Clinical Investigation at 8:00 AM after a 12-hour overnight fast. A stool sample from each subject was also collected. Exclusion criteria included known hepatic diseases (except for NAFLD), alcohol consumption, smoking, and medication use. Written parental informed consent and written child assent were obtained from all participants. Yale University Human Investigation Committee approved the study.

### Imaging

Measurement of liver fat content was performed by MRI using the 2-point Dixon method, and hepatic fat fraction (HFF) was calculated from the mean pixel signal intensity data. MRI studies were performed on a GE or Siemens Sonata 1.5 Tesla system.

### Genotyping

Genomic DNA was extracted from peripheral blood leukocytes from 8 cc of whole blood using the guanidine HCl DNA extraction protocol. To genotype the rs738409 single-nucleotide polymorphism, the following pair of primers was used: forward = 5′-GCCCTGCTCACTTGGAGAAA-3′ and reverse = 5′-TGAAAGGCAGTGAGGCATGG-3′. Polymerase chain reaction (PCR) was performed using an annealing temperature of 65.0°C and PCR products were analyzed by automatic sequencing at the Yale W.M. Keck Facility.

### Microbial DNA extraction and 16S rRNA gene sequencing

Samples were processed in 2 separate batches; the larger subset (60 samples) in 2016 and the smaller subset (13 samples) in 2019. DNA was extracted using the MoBio PowerMag Soil 96-well kit (MoBio Laboratories, Carlsbad, California) for the larger subset of samples. The smaller subset samples were extracted using the DNeasy PowerSoil Kit (Qiagen, Hilden, Germany), as the MoBio kit was no longer available. Based on available information, the 2 kits only differ in the method of purification. The lysis step in both methods is identical, including the size of beads for mechanical lysis and the composition of the lysis buffer.

Using either the MoBio PowerMag Soil 96 well kit or the Qiagen DNeasy PowerSoil kit, DNA was extracted from 0.25 g of fecal sample. DNA extracts were quantified using the Quant-iT PicoGreen kit (Invitrogen, ThermoFisher Scientific) for the larger subset of samples and the Qubit High Sensitivity kit (Invitrogen, ThermoFisher Scientific) for the smaller subset. The V4 region of the 16S rRNA gene was amplified using primers described in Caporaso et al (21) with dual-indexed adapters for indexing and 30 ng of input DNA. Samples were amplified in triplicate using Phusion High-Fidelity PCR master mix (New England BioLabs, Ipswich, MA) with the addition of 1 µg BSA (New England BioLabs, Ipswich, MA). The PCR reaction was incubated at 95 °C for 3.5 minutes, 30 cycles of 30 seconds at 95 °C, 30 seconds at 50 °C and 90 seconds at 72 °C, followed by final extension at 72 °C for 10 minutes. PCR triplicate reactions were combined for quantification and visualization using the QIAxcel DNA Fast Analysis kit (Qiagen, Hilden, Germany). PCR products were pooled based on the concentration of DNA from 250 to 400 base pairs using the EpMotion liquid handling robot (Eppendorf, Hamburg, Germany) and cleaned using the Gene Read Size Selection kit (Qiagen, Hilden, Germany) per the manufacturer’s protocol. The cleaned pool was diluted to 4 nM and sequenced on a MiSeq (Illumina, San Diego, CA) using the v2 2 × 250 base pair kit and following manufacturer’s protocol.

The 16S rRNA V4 gene sequence data was demultiplexed through BaseSpace (Illumina, San Diego, CA). Paired-end data was then imported into QIIME 2 2019.4 (22) and reads were filtered, denoised, and dereplicated using the dada2 denoise-paired plugin (23). Sequences were taxonomically classified and compiled using the feature-classifier and taxa (24-29) plugins. Taxonomy was assigned using a pre-trained Naïve Bayes classifier based on the Greengenes 13_8 99% Operational Taxonomic Units (OTUs) database (30) trimmed to only include the region bound by the 515F/806R primer pair. Default Qiime2 processing parameters were used throughout the workflow except where otherwise noted (refer to code availability section).

Data was imported into RStudio 3.5.2 using the qiime2R plugin (31) and analyzed using the phyloseq (32) and pheatmap (33) packages. Phyloseq was used for alpha diversity calculations and a rarefaction depth of 10 000 reads was chosen. The bacterial abundance heatmap was generated by pruning genera with fewer than 250 reads summed across all samples and then normalizing read depth to 10 000 reads for all samples. These values were then log transformed and plotted using pheatmap. Controls were run according to Benjamino et al (34). Briefly, a negative extraction control and PCR control were run and no reads were retained following denoising. Mock community controls (Zymo, Irvine, CA) were also run and composition was as expected.

### Statistical analyses

The distribution of continuous variables was evaluated for skewness and kurtosis, and by using a histogram with the normal curve imposed. When appropriate, nonparametric tests were utilized. When needed, data were transformed into ranks to reduce the influence of outliers. Subjects were divided into 2 groups, HFF <5.5% (n = 29) and HFF ≥5.5% (n = 44). Student’s *t* tests, Mann-Whitney U tests, and chi-square tests were utilized to evaluate differences in the characteristics of the 2 groups. The following characteristics were compared using Student’s *t* tests (parametric) between the 2 HFF groups: age, height, weight, BMI, BMI z-score, 2-hour glucose, cholesterol, high-density lipoprotein (HDL), low-density lipoprotein (LDL), HFF, and visceral fat. Mann-Whitney U tests (nonparametric) were utilized for the following characteristics: fasting glucose, fasting insulin, whole-body insulin sensitivity index (WBISI), triglycerides, alanine aminotransferase (ALT), and subcutaneous fat. Chi-square tests were utilized to evaluate differences between the 2 groups in sex, race, and *PNPLA3* genotype. When appropriate, data are presented as mean (standard deviation).

Shannon-Wiener diversity index values, an alpha diversity measure that accounts for the number of species and evenness of their abundance, were calculated for all stool samples using the R package phyloseq. The values were compared between the 2 HFF groups using Welch’s *t* test, to account for unequal variances between the 2 groups. The abundance of each bacterial phyla and genera was compared between the 2 HFF groups using Student’s *t* test or Mann-Whitney U test. Phyla where a phylum did not reach 1% abundance in at least one sample were excluded from the analysis. Therefore, 8 out of 13 phyla were included in the analysis. Genera in which a genus did not reach 1% abundance in at least one sample were not included in the analysis. Thus, 51 out of 125 phyla were included in the analysis. The abundance of each phylum and genus was compared between the 2 groups using Mann-Whitney U test for all phyla and genera, except for Firmicutes and *Faecalibacterium*, in which Student’s *t* tests were used.

Simple linear regression, with HFF as the outcome, was conducted for each phylum and genus that was significantly different in abundance between the 2 HFF groups. While controlling for *PNPLA3* genotype, race, and BMI z-score, multiple linear regression was conducted for each phylum and genus that was significantly different in abundance between the 2 HFF groups to determine if the respective phylum/genus was found to be predictive of HFF. For a phylum or genus that was not normally distributed, abundance values were ranked and the ranked abundance values were used in the regression model. Additionally, each multiple linear regression model was checked for normality using a Predicted Probability plot and for absence of multicollinearity using variance inflation factor values.

## Results

We compared characteristics between the groups with and without NAFLD (Table 1). There were no significant differences between the 2 groups in terms of age (*P* = 0.616), sex (*P* = 0.316), height (*P* = 0.166), and BMI (*P* = 0.061). The groups significantly differed in race (*P* = 0.008), with a greater proportion of African American participants in the group without NAFLD. Despite both groups having BMI greater than 95th percentile, the group with NAFLD was found to have greater weight (*P* = 0.047) and BMI z-score (*P* = 0.008), compared with the group without NAFLD. In regards to glucose metabolism, the groups had comparable fasting glucose (*P* = 0.450) and 2-hour glucose (*P* = 0.442) values, but the group with NAFLD had increased fasting insulin (*P* < 0.001) and decreased WBISI (*P* < 0.001). The lipid profile between the 2 groups was similar in total cholesterol (*P* = 0.468) and LDL (*P* = 0.948); however, the group with NAFLD had lower HDL (*P* = 0.014) and higher triglycerides (*P* < 0.001). Participants with NAFLD displayed significantly higher HFF (*P* < 0.001) and ALT (*P* < 0.001). Comparing body fat distribution between the 2 groups, they did not differ in subcutaneous fat composition (*P* = 0.253), but visceral fat composition was higher in the group with NAFLD (*P* < 0.001). The groups differed in the *PNPLA3* genotype (*P* < 0.001), with the GG genotype (homozygous for risk allele) present only in the group with NAFLD.

**Table 1.** Characteristics of the Study Population Grouped by HFF

	HFF <5.5% (n= 29)	HFF ≥5.5% (n= 44)	*P*
**DEMOGRAPHICS**			
**Age (years)**	12.9 (2.8)	13.3 (3.2)	0.616
**Sex**			0.316
Male	13 (44.8%)	25 (56.8%)	
Female	16 (55.2%)	19 (43.2%)	
**Race**			**0.008**
Caucasian	7 (24.1%)	17 (38.6%)	
African American	9 (31.0%)	2 (4.5%)	
Hispanic	13 (44.8%)	25 (56.8%)	
**ANTHROPOMETRICS**			
**Height (cm)**	158.1 (12.6)	162.5 (13.2)	0.166
**Weight (kg)**	83.1 (24.4)	95.4 (25.9)	**0.047**
**BMI (kg/m** ^**2**^)	32.5 (6.8)	35.5 (6.4)	0.061
**BMI z-score**	2.2 (0.33) (n=29)	2.4 (0.32) (n=43)	**0.008**
**GLUCOSE METABOLISM**			
**Fasting glucose (mg/dL)**	90.6 (5.8)	92.4 (9.8)	0.450
**Fasting insulin (μU/mL)**	24.6 (9.0) (n=29)	46.3 (26.8) (n=43)	**<0.001**
**2-hour glucose (mg/dL)**	120.7 (23.8)	125.9 (29.9)	0.442
**WBISI**	2.1 (0.94) (n=29)	1.3 (0.75) (n=43)	**<0.001**
**LIPID PROFILE**			
**Total Cholesterol (mg/dL)**	148.0 (29.4) (n=29)	152.9 (26.7) (n=43)	0.468
**HDL Cholesterol (mg/dL)**	46.6 (8.1) (n=29)	41.1 (9.6) (n=43)	**0.014**
**LDL Cholesterol (mg/dL)**	86.1 (26.2) (n=29)	86.5 (21.8) (n=43)	0.948
**Triglycerides (mg/dL)**	76.2 (39.2) (n=29)	133.1 (83.4) (n=43)	**<0.001**
**LIVER FUNCTION**			
**Hepatic Fat Fraction (%)**	1.4 (1.6)	19.6 (11.3)	**<0.001**
**ALT (U/L)**	17.9 (7.6) (n=28)	46.5 (32.2) (n=41)	**<0.001**
**BODY FAT DISTRIBUTION**			
**Visceral (cm** ^**2**^)	53.0 (21.7)	87.3 (34.0)	**<0.001**
**Subcutaneous (cm** ^**2**^)	541.1 (270.9)	567.8 (186.3)	0.253
**GENETIC COMPOSITION**			
***PNPLA3* rs738409**			**<0.001**
CC	16 (55.2%)	13 (29.5%)	
CG	13 (44.8%)	12 (27.3%)	
GG	0 (0%)	19 (43.2%)	

Statistically significant P values are indicated in bold.

Abbreviations: ALT, alanine aminotransferase; BMI, body mass index; HDL, high-density lipoprotein; LDL, low-density lipoprotein; *PNPLA3*, Patatin-like phospholipase 3 gene; WBISI, whole-body insulin sensitivity index.

The distribution of phyla and genera per NAFLD status for each participant is shown in Supplementary Figure 1 (35, 43). In particular, the figure shows the abundance of phyla and genera among stool samples. NAFLD status is denoted at the top with red indicating the presence of NAFLD and green indicating the absence of NAFLD (yes and no, respectively). Genera are grouped by phylum and phyla are denoted on the left with colored bars. The heatmap is displaying normalized abundance data on a log scale with blue indicating lower abundance and red indicating higher abundance.

**Figure 1.**
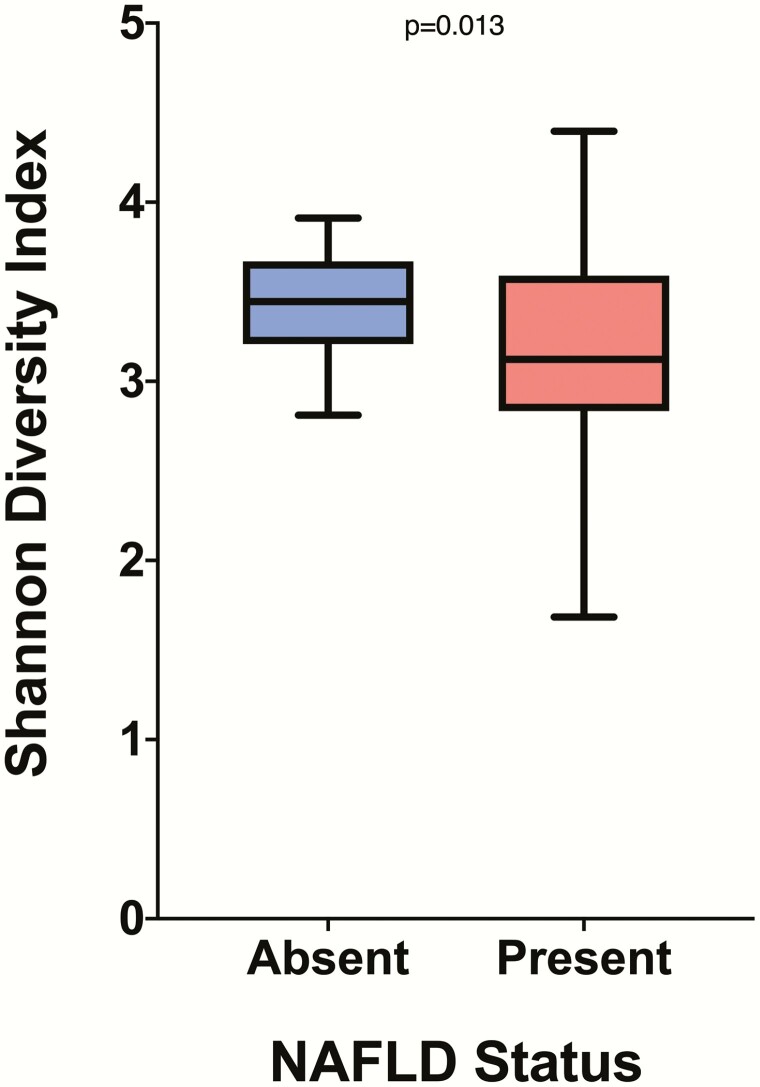
**Alpha diversity in NAFLD absent and present groups.** The figure shows a significant difference (*P* = 0.013) in Shannon diversity index values between the group without NAFLD, in blue, and the group with NAFLD, in red. Boxplots are presented in Tukey style. The groups were compared using Welch’s *t* test.

**Figure 2.**
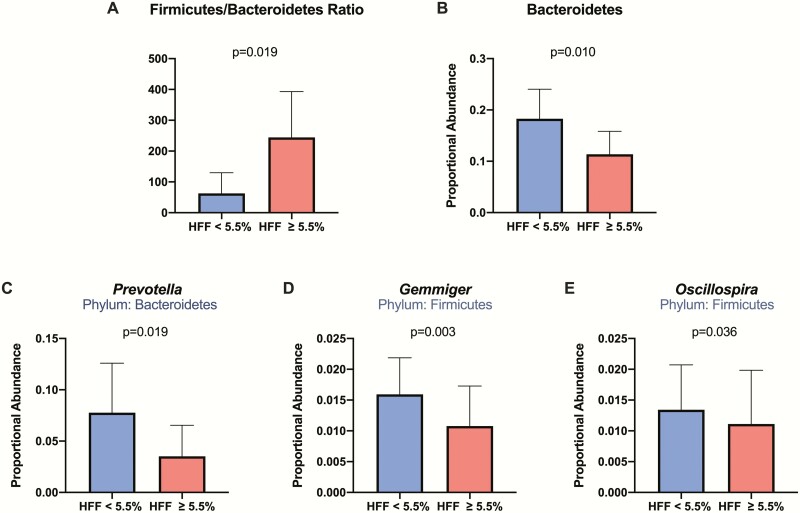
**Difference in phylum/genus abundance in HFF groups.** The figure shows the mean with SD in Firmicutes/Bacteroidetes (F/B) ratio (**A**), Bacteroidetes abundance (**B**), *Prevotella* abundance (**C**), *Gemmiger* abundance (**D**), and *Oscillospira* abundance (**E**) observed in the 2 HFF groups. The group without NAFLD is shown in blue and the group with NAFLD is shown in red. The 2 groups were compared using Mann-Whitney U test.

Comparing the Shannon-Wiener diversity index values between the 2 HFF groups revealed that subjects with NAFLD have decreased (*P* = 0.013) bacterial diversity compared with those without NAFLD (Fig. 1). The NAFLD group had a Shannon-Wiener diversity index mean of 3.174, whereas the group without NAFLD had a Shannon-Wiener diversity index mean of 3.428. There was a significantly higher Firmicutes to Bacteroidetes (F/B) ratio (*P* = 0.019) in participants with NAFLD (Fig. 2). From the 8 phyla abundances that were compared between groups, we found lower proportional abundance of Bacteroidetes (*P* = 0.010) in the group with NAFLD. Among the 51 examined genera, *Prevotella* (*P* = 0.019), *Gemmiger* (*P* = 0.003), and *Oscillospira* (*P* = 0.036) were lower in proportional abundance in the group with NAFLD, compared with the group without NAFLD.

The simple linear regression model with HFF as dependent variable and Firmicutes/Bacteroidetes (F/B) ratio as independent variable was significant and explained 15.3% of the variation in HFF (Table 2). The multiple linear regression model that was conducted to determine if F/B ratio was predictive of HFF, while controlling for *PNPLA3* genotype, race, and BMI z-score, found that for every unit increase of the rank of F/B ratio, there is a 0.179 increase in HFF (Table 3). This model with F/B ratio, *PNPLA3* genotype, race, and BMI z-score as independent variables explained 29.9% of the variation in HFF.

**Table 2.** Simple Linear Regression With HFF as Dependent Variable

Outcome: HFF^a^		R squared: 0.153	
** Model Variables**	**B (Unstandardized)**	**β (Standardized)**	***P***
** (Intercept)**	3.799		0.173
** Ranked F/B Ratio**	0.232	0.391	**0.**001
Outcome: HFF		R squared: 0.148	
** Model Variables**	**B (Unstandardized)**	**β (Standardized)**	***P***
** (Intercept)**	20.845		<0.001
** Ranked Bacteroidetes**	-0.229	-0.385	**0.**001
Outcome: HFF^a^	R squared: 0.060		
** Model Variables**	**B (Unstandardized)**	**β (Standardized)**	***P***
** (Intercept)**	17.858		<0.001
** Ranked *Prevotella***	-0.148	-0.246	**0.**036
Outcome: HFF		R squared: 0.069	
** Model Variables**	**B (Unstandardized)**	**β (Standardized)**	***P***
** (Intercept)**	18.146		<0.001
** Ranked *Gemmiger***	-0.156	-0.262	**0.**025
Outcome: HFF^a^		R squared: 0.103	
** Model Variables**	**B (Unstandardized)**	**β (Standardized)**	***P***
** (Intercept)**	19.458		<0.001
** Ranked *Oscillospira***	-0.191	-0.321	**0.006**

The table shows that simple linear regression with HFF as the outcome was conducted separately for F/B ratio, Bacteroidetes, *Prevotella, Gemmiger, and Oscillospira*. Statistically significant *P* values are indicated in bold.

Abbreviation: F/B ratio, Firmicutes to Bacteroidetes ratio; HFF, hepatic fat fraction.

^a^The model significantly predicts HFF (P < 0.05).

**Table 3.** Multiple Linear Regression Models

Outcome: HFF^a^	R squared: 0.299		
Model Variables	B (Unstandardized)	β (Standardized)	*P*
**(Intercept)**	-10.979		0.262
**Ranked F/B Ratio**	0.179	0.306	**0.006**
***PNPLA3* rs738409**	3.173	0.204	0.082
**BMI z-score**	6.842	0.184	0.087
**Caucasian**	reference		
**African American**	-8.330	-0.243	0.048
**Hispanic**	-2.077	-0.084	0.475
Outcome: HFF^a^	R squared: 0.292		
Model Variables	B (Unstandardized)	β (Standardized)	*P*
**(Intercept)**	2.143		0.842
**Ranked Bacteroidetes**	-0.169	-0.289	**0.009**
***PNPLA3* rs738409**	3.530	0.227	0.052
**BMI z-score**	6.522	0.175	0.107
**Caucasian**	reference		
**African American**	-7.571	-0.220	0.072
**Hispanic**	-1.919	-0.078	0.511
Outcome: HFF^a^	R squared: 0.245		
Model Variables	B (Unstandardized)	β (Standardized)	*P*
**(Intercept)**	-4.003		0.708
**Ranked *Prevotella***	-0.106	-0.177	0.111
***PNPLA3* rs738409**	4.176	0.268	**0.024**
**BMI z-score**	7.658	0.206	0.065
**Caucasian**	reference		
**African American**	-7.147	-0.208	0.099
**Hispanic**	-0.928	-0.038	0.759
Outcome: HFF^a^	R squared: 0.260		
Model Variables	B (Unstandardized)	β (Standardized)	*P*
**(Intercept)**	-9.084		0.365
**Ranked *Gemmiger***	-0.134	-0.226	**0.048**
***PNPLA3* rs738409**	4.429	0.284	**0.015**
**BMI z-score**	9.851	0.265	**0.018**
**Caucasian**	reference		
**African American**	-4.566	-0.133	0.303
**Hispanic**	-0.224	-0.009	0.941
Outcome: HFF^a^	R squared: 0.268		
Model Variables	B (Unstandardized)	β (Standardized)	*P*
**(Intercept)**	1.515		0.892
**Ranked *Oscillospira***	-0.142	-0.239	**0.032**
***PNPLA3* rs738409**	4.440	0.285	**0.015**
**BMI z-score**	6.050	0.163	0.148
**Caucasian**	reference		
**African American**	-7.085	-0.206	0.097
**Hispanic**	-2.079	-0.084	0.484

The table shows that multiple linear regression with HFF as the outcome was conducted separately for F/B ratio, Bacteroidetes, *Prevotella, Gemmiger, and Oscillospira*, while also including *PNPLA3* genotype, BMI z-score, and race in each model. Statistically significant P values are indicated in bold.

Abbreviations: BMI, body mass index; F/B ratio, Firmicutes to Bacteroidetes ratio; *HFF*, hepatic fat fraction; *PNPLA3*, Patatin-like phospholipase 3 gene.

^a^The model significantly predicts HFF (P < 0.05).

The simple linear regression model with HFF as dependent variable and Bacteroidetes as independent variable was significant and explained 14.8% of the variation in HFF (Table 2). The multiple linear regression model that was conducted to determine if Bacteroidetes abundance was predictive of HFF, while controlling for *PNPLA3* genotype, race, and BMI z-score, found that as Bacteroidetes abundance increased, HFF decreased (Table 3). Bacteroidetes, *PNPLA3* genotype, race, and BMI z-score explained 29.2% of the variation in HFF.

The simple linear regression model with HFF as dependent variable and *Prevotella* as independent variable was significant and explained 6.0% of the variation in HFF (Table 2). When controlling for *PNPLA3* genotype, race, and BMI z-score, *Prevotella* abundance was no longer predictive of HFF; however, *PNPLA3* genotype was a significant predictor of the outcome (Table 3).

The simple linear regression model with HFF as dependent variable and *Gemmiger* as independent variable was significant and explained 6.9% of the variation in HFF (Table 2). The multiple linear regression model that was conducted to determine if *Gemmiger* was predictive of HFF, while controlling for *PNPLA3* genotype, race, and BMI z-score, found that as *Gemmiger* abundance increased, HFF decreased (*P* = 0.048) (Table 3). In this model, *PNPLA3* genotype and BMI z-score were also predictors of HFF. *Gemmiger*, *PNPLA3* genotype, race, and BMI z-score explained 26.0% of the variation in HFF.

The simple linear regression model with HFF as dependent variable and *Oscillospira* as independent variable was significant and explained 10.3% of the variation in HFF (Table 2). The multiple linear regression model that was conducted to determine if *Oscillospira* was predictive of HFF, when controlling for *PNPLA3* genotype, race, and BMI z-score, noted that as *Oscillospira* abundance increased, HFF decreased (*P* = 0.032) (Table 3). In this model, *PNPLA3* genotype was also a predictor of the dependent variable. *Oscillospira*, *PNPLA3* genotype, race, and BMI z-score explained 26.8% of the variation in HFF.

**Figure 3.**
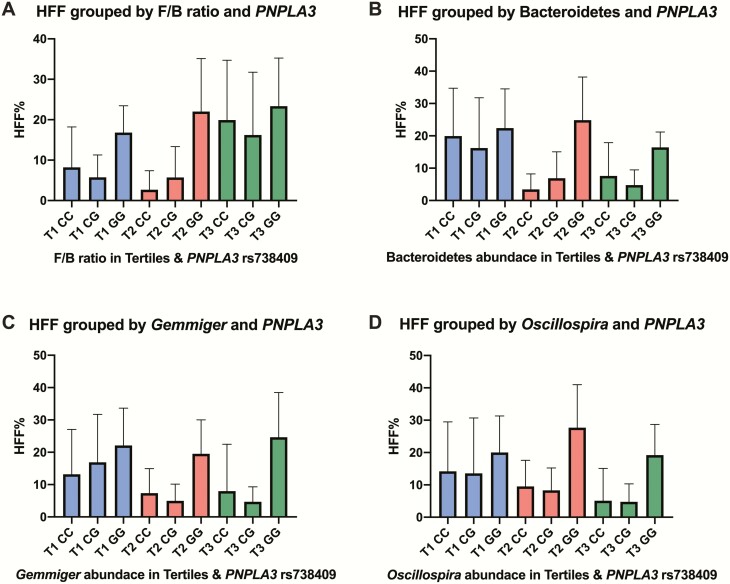
**Effect of *PNPLA3* variant and bacteria on HFF.** The figure shows the additive effect of *PNPLA3* rs738409 genotype and F/B ratio (**A**), Bacteroidetes (**B**), *Gemmiger* (**C**), and *Oscillospira* (**D**) on hepatic fat fraction (HFF). Bacterial abundance was categorized in tertiles, from smallest (blue) to largest (green) abundance, T1 to T3. Data are shown as mean and SD.

To illustrate the effect of the bacteria and the *PNPLA3* variant on HFF, we categorized the bacterial abundances in tertiles, from smallest to largest (Fig. 3). Participants with *PNPLA3* genotype CC (risk allele absent) and third tertile (highest abundance) of *Gemmiger* had a mean HFF value of 7.98% (14.5%), whereas participants with *PNPLA3* genotype GG (homozygous for risk allele) and first tertile (lowest abundance) of *Gemmiger* had a mean HFF value of 22.1% (11.5%). Participants with *PNPLA3* genotype CC and third tertile (highest abundance) of *Oscillospira* had a mean HFF value of 5.10% (10.0%), whereas participants with *PNPLA3* genotype GG and first tertile (lowest abundance) of *Oscillospira* had a mean HFF value of 20.03% (11.3%).

## Discussion

Our data substantiate recent findings that NAFLD is associated with changes of the intestinal microbiota. Subjects with NAFLD were found to have decreased bacterial diversity and richness compared with those without NAFLD. Additionally, we observed a significantly higher Firmicutes to Bacteroidetes ratio and lower abundance of Bacteroidetes, *Prevotella*, *Gemmiger*, and *Oscillospira* in the group with NAFLD, compared with the group without NAFLD. When controlling for *PNPLA3* genotype, race, and BMI z-score, regression analyses demonstrated that F/B ratio, Bacteroidetes, *Gemmiger*, and *Oscillospira* were all individually associated with HFF. These regression models with *PNPLA3* genotype, race, BMI z-score, and F/B ratio, Bacteroidetes, *Gemmiger*, or *Oscillospira* explained over 20% of the variation in HFF. We also discovered an additive effect on HFF from *PNPLA3* and *Gemmiger*, and *PNPLA3* and *Oscillospira.*

Our results on fecal microbiome alpha diversity agree with those of Del Chierico et al (16) and Schwimmer et al (20), where participants with NAFLD also showed decreased bacterial diversity compared with participants without NAFLD (*P* < 0.05). Since the gut microbiota protects the host from colonization and invasion of other pathogens, having a greater bacterial diversity may better protect against injurious colonization of other pathogens and prevent bacteria and toxins from gaining access to the portal circulation and releasing inflammatory cytokines in the liver. For over a decade, studies of lean and obese mice have proposed that gut microbiota dysbiosis may increase energy harvest from the diet and alter how this energy is utilized and stored (18, 36). In pediatrics, the gut microbiota of obese youth has been observed to have a higher capacity to oxidize carbohydrates, as compared with lean youth (37). Therefore, it is a possibility that the bacteria found in obese children with NAFLD have greater pathogenic or opportunistic tendencies and outcompeted bacteria that were more beneficial to the host. For example, in our obese participants with NAFLD, we observed a decreased abundance of Bacteroidetes, which has repeatedly been found in greater abundance in lean subjects compared with obese subjects (18, 19).

We observed a significantly lower abundance of *Oscillospira* in the group with NAFLD, compared with the group without NAFLD, which is consistent with the findings from the Michail et al (17) study. In the study, an *Oscillospira* decrease coupled to up-regulation of 2-butanone was identified as signature of NAFLD onset, although this was based on comparisons with lean controls. While the abundance of *Oscillospira* was higher in obese children without NAFLD relative to those with NAFLD, the study does not disclose if this difference was significant. Our multiple linear regression model, which controlled for *PNPLA3* genotype, race, and BMI z-score, showed that both *Oscillospira* and *PNPLA3* genotype were predictive of HFF. Thus, there is an additive effect between *Oscillospira* and *PNPLA3* rs738409 variant in conveying susceptibility to NAFLD, in which both factors independently affect HFF.

Unlike the studies by Del Chierico et al (16) and Michail et al (17), we observed a statistically significant difference in the F/B ratio between the groups with and without NAFLD. This could be due to having a greater sample size to make statistical inferences. Additionally, Del Chierico et al (16) reported a higher abundance of Bacteroidetes in children with NAFL compared with obese children, similar to our observations, but the difference was not statistically significant. The differences in results could also be explained by our use of amplicon sequence variants (ASVs) as opposed to OTUs, which were utilized in Del Chierico et al (16). It has been suggested that ASVs provide higher resolution and better accuracy than OTUs, thus, in some cases, allowing for additional genus and species level assignments to be made (23). Additionally, the choice of hypervariable region has been shown to have a significant effect on accurately determining diversity and resolution for microbiome profiling studies (38, 39), making it difficult to compare results when different regions are used. Primer biases can also play a role when different hypervariable regions are targeted, excluding or overrepresenting particular taxa (40). Michail et al (17) used a DNA microarray for 16S rRNA profiling, but this approach has its own limitations, namely the fact that the microarray itself is based on known sequence variants. This raises the potential for missing diversity that would otherwise be detected by next-generation sequencing (41). Additionally, environmental factors specific to our study population, such as diet, could account, at least in part, for our findings.

While Schwimmer et al (20) also found a significant difference in the abundance of Bacteroidetes between the 2 groups, the direction of the relationship was opposite to that of our results. The researchers observed greater abundance of Bacteroidetes in obese children with NAFLD compared with those without NAFLD, while we observed higher abundance of Bacteroidetes in the group without NAFLD. Additionally, Schwimmer et al (20) found that high abundance of *Prevotella copri* was associated with more severe fibrosis, while our study noticed lower abundance of *Prevotella* in the group with NAFLD, compared with the group without NAFLD. When we accounted for *PNPLA3* genotype, *Prevotella* was no longer predictive of NAFLD status. The discrepancy between our findings and that of Schwimmer et al (20) could possibly be explained by differences in the methods of stool DNA extraction and downstream analysis. In their study, the stool samples were placed in RNAlater and rocked overnight at 4 °C and stored at −80 °C until isolation (20). In contrast, our samples were collected and directly stored at −20 °C until isolation. A study by Bahl et al (42) revealed that in 7 of 9 cases, the Firmicutes to Bacteroidetes ratio was significantly higher in fecal samples that had been frozen compared with identical samples that had not. According to the researchers, in order to make comparisons within a study, all samples should be frozen in a similar manner, which was the case with our study. The Schwimmer et al (20) study also used the V1-V3 hypervariable region of the 16S rRNA gene and clustered sequences into OTUs, as described above, which may contribute to some differences. Because of the differences in DNA extraction and in the hypervariable region used, comparisons of the gut microbiome between these research projects may not be feasible. In addition, direct comparisons are further complicated if the taxa of interest are less abundant.

This study provides important insights regarding the interaction of the microbiome and NAFLD. A diverse gut microbiome correlates with reduced prevalence of NAFLD, providing further support for microbial community richness improving host health. Our results show that the F/B ratio and certain bacterial genera have statistically significant associations with HFF and NAFLD status, even when controlling for factors associated with NAFLD, including *PNPLA3* rs738409. Additional information, such as liver biopsies and shotgun metagenome data for more patients, would strengthen the current study and include limitations of our study; this will be pursued in the future. Another limitation of this study is its descriptive nature; thus, future directions include testing the causal relationship of intestinal bacteria on NAFLD by modifying bacterial abundance and composition with the appropriate probiotics as an adjunct to weight loss and/or dietary changes while measuring HFF at baseline and after intervention. While descriptive, two important strengths of our study are the thorough phenotype that allows consideration of metabolic and genetic factors and the young age of the studied population.

Our findings agree with the notion that gut microbiota composition and diversity could potentially be used to detect susceptibility to NAFLD, which has been mentioned in previous studies (16, 17, 20). Notably, this tool may be used in conjunction with *PNPLA3* rs738409 genotype. Additionally, we identified specific bacterial candidates for testing causal relationships and that may be beneficial as probiotics in the treatment of NAFLD. Overall, our data support previous experimental observations while also providing the foundation for testing novel hypotheses in future studies.

## Data Availability

Data for the larger subset of samples can be found in the NCBI SRA database under project ID PRJNA328258. Data for the smaller subset of samples can be found in the NCBI SRA database under project ID PRJNA606577 (43). ***Code availability:*** Source code, including Qiime2 processing commands and R commands used, is available at https://github.com/joerggraflab/Code-for-Kravetz-Testerman-2020(35).

## Abbreviations

ALT: alanine aminotransferase
ASV: amplicon sequence variant
BMI: body mass index
F/B ratio: Firmicutes to Bacteroidetes ratio
HDL: high-density lipoprotein
LDL: low-density lipoprotein
MRI: magnetic resonance imaging
MRI: magnetic resonance imaging
NAFLD: nonalcoholic fatty liver disease
OGTT: oral glucose tolerance test
OTU: operational taxonomic unit
PCR: polymerase chain reaction
PNPLA3: Patatin-like phospholipase 3 gene
WBISI: whole-body insulin sensitivity index

## Additional Information

***Disclosure Summary:*** The authors have nothing to disclose.
